# Supplementary material for: Quantum magnetisms in uniform triangular lattices Li2AMo3O8 (A = In, Sc)
Source: Sci Rep. 2019 Feb 12;9:1826. doi: 10.1038/s41598-018-36123-7 (PMC6372599; doi:10.1038/s41598-018-36123-7)
Supplement: Supplementary file 1 — Supplementary Information [file 41598_2018_36123_MOESM1_ESM.pdf]

Supplementary Information for

Quantum magnetisms in uniform triangular lattices $Li_2AMo_3O_8$ (A = In, Sc)

Kazuki Iida[1,*], Hiroyuki Yoshida[2], Hirotaka Okabe[3], Naoyuki Katayama[4],
Yuto Ishii[2], Akihiro Koda[3,5], Yasuhiro Inamura[6], Naoki Murai[6],
Motoyuki Ishikado[1], Ryosuke Kadono[3,5] & Ryoichi Kajimoto[6]

[1] *Neutron Science and Technology Center, Comprehensive Research Organization for Science and Society (CROSS), Tokai, Ibaraki 319-1106, Japan*

[2] *Department of Physics, Faculty of Science, Hokkaido University, Sapporo, Hokkaido 060-0810, Japan*

[3] *Institute of Materials Structure Science, High Energy Accelerator Research Organization (KEK), Tokai, Ibaraki 319-1106, Japan*

[4] *Department of Applied Physics, Nagoya University, Nagoya, Aichi 464-8603, Japan*

[5] *Department of Materials Structure Science, Sokendai (The Graduate University for Advanced Studies), Tsukuba, Ibaraki 305-0801, Japan*

[6] *J-PARC Center, Japan Atomic Energy Agency (JAEA), Tokai, Ibaraki 319-1195, Japan*

* k_iida@cross.or.jp

Synchrotron X-ray diffraction on $Li_2InMo_3O_8$ and $Li_2ScMo_3O_8$

Synchrotron X-ray diffraction measurements on $Li_2InMo_3O_8$ and $Li_2ScMo_3O_8$ were performed at the BL5S2 beamline equipped at Aichi Synchrotron Radiation Center, Japan. Measurements were performed at 300 K with synchrotron radiation of λ = 0.6520 Å. Rietveld analysis was performed using RIETAN-FP [S1]. At first, Rietveld refinement was performed by assuming small substitutions of In (Sc) by Li and Li by In (Sc) with keeping the composition ratio, Li : In (Sc) : Mo : O = 2 : 1 : 3 : 8 for both samples. As a result, we found that the substitution amount is zero within errors for both samples. Therefore, we exhibit the Rietveld refinement results obtained without assuming any substitutions in Figs. S1 and S2 for $Li_2InMo_3O_8$ and $Li_2ScMo_3O_8$, respectively. Obtained structural parameters are summarized in Tables S1 and S2.

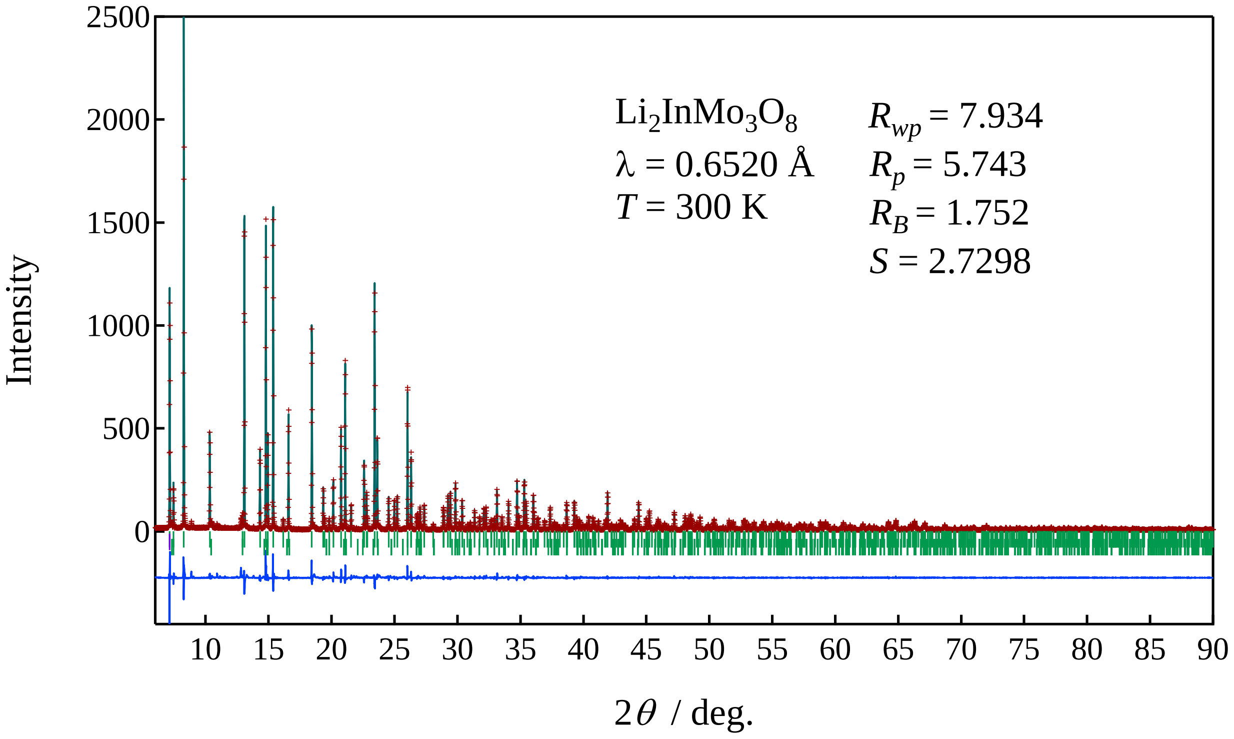

Figure S1. Rietveld refinement of $Li_2InMo_3O_8$. The experimental (plus) and fitted (line) X-ray diffraction patterns at 300 K are shown. Vertical bars under the diffraction peaks show the Bragg peak reflection positions. The plots under the bars represent residues. Rietveld analysis was performed by assuming the presence of impurity phase of $LiInMo_3O_8$ with the molar ratio of 1.6%. Some small amount of unknown impurities also exist, which could not be identified in the present analysis. The crystallographic parameters calculated from these refinements are presented in Table S1.

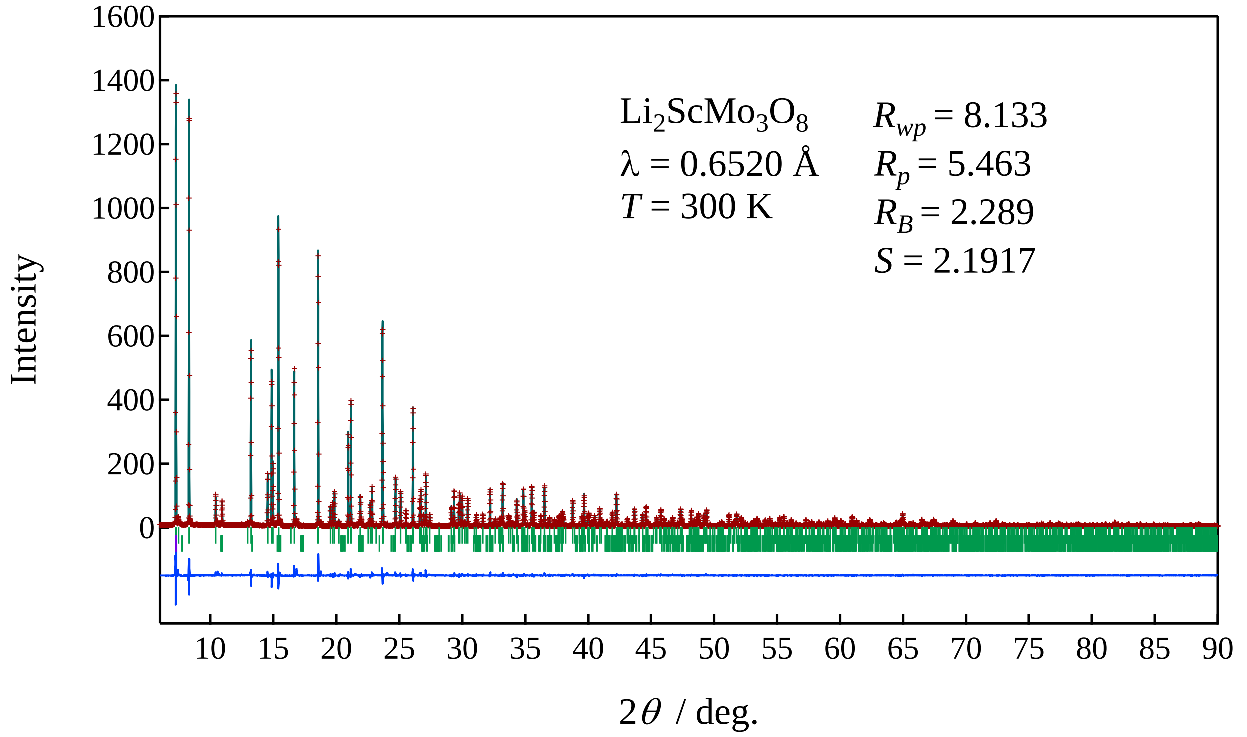

Figure S2. Rietveld refinement of $Li_2ScMo_3O_8$. The experimental (plus) and fitted (line) X-ray diffraction patterns at 300 K are shown. Vertical bars under the diffraction peaks show the Bragg peak reflection positions. The plots under the bars represent residues. Rietveld analysis was performed by assuming the presence of impurity phase of MoO_2 with the molar ratio of 9.8%. The crystallographic parameters calculated from these refinements are presented in Table S2.

Table S1. Structural parameters of $Li_2InMo_3O_8$.

$P6_3mc$, $a = b$ = 5.78701(4) Å, c = 10.45632(6) Å						
	site	g	x	y	z	B (Å^2)
Li1	2*a*	1.0	0	0	0.16959	1.600
Li2	2*b*	1.0	1/3	2/3	0.06176	1.600
In1	2*b*	1.0	1/3	2/3	0.68931(64)	0.222(9)
Mo1	6*c*	1.0	0.18656(4)	0.81344	0.40477(63)	0.110(6)
O1	6*c*	1.0	0.84255(39)	0.15747	0.30102(80)	0.370(46)
O2	2*a*	1.0	0	0	0	0.489(97)
O3	2*b*	1.0	1/3	2/3	0.27118(91)	0.151(78)
O4	6*c*	1.0	0.51306(37)	0.48686	0.03307(76)	0.386(51)

Table S2. Structural parameters of $Li_2ScMo_3O_8$.

$P6_3mc$, $a = b$ = 5.77702(3) Å, c = 10.29320(5) Å						
	site	g	x	y	z	B (Å^2)
Li1	2*a*	1.0	0	0	0.16959	1.600
Li2	2*b*	1.0	1/3	2/3	0.06176	1.600
Sc1	2*b*	1.0	1/3	2/3	0.67641(57)	0.405(21)
Mo1	6*c*	1.0	0.18644(3)	0.81356	0.39324(54)	0.138(5)
O1	6*c*	1.0	0.83868(30)	0.16132	0.28474(62)	0.298(38)
O2	2*a*	1.0	0	0	0	0.679(85)
O3	2*b*	1.0	1/3	2/3	0.25463(76)	0.140(67)
O4	6*c*	1.0	0.51407(29)	0.48593	0.02433(59)	0.383(39)

Theoretical calculations of local magnetic fields in $Li_2ScMo_3O_8$

Identification of muon stopping sites in materials is essential to discuss the internal magnetic fields of them. Here, we explain how we identify the muon site and calculate local magnetic fields in $Li_2ScMo_3O_8$.

Figure S3 displays the crystal structure of $Li_2ScMo_3O_8$ with isosurfaces of electrostatic potential which are obtained by density functional theory (DFT) calculation using the Vienna *ab*-initio simulation package (VASP) [S2]. Implanted muons tend to reside the local minima of electrostatic potential (yellow portions in Fig. S3) which locate near oxygen O1 (0.5193, 0.4807, 0.0319), O2 (0.8429, 0.1571, 0.2862), and O3 (0, 0, 0). Note that there are no local minima near O4 (1/3, 0.5, 0.2585). Similar electrostatic potential was obtained for $Li_2InMo_3O_8$.

We assumed three possible oxygen-muon bonds O1-μ1, O2-μ2, and O3-μ3 with the bond length of 1.2 Å which extend to the closest local minima. Table S3 shows the local magnetic fields δ_μ and nuclear dipolar field widths Δ_μ in the possible muon sites calculated by the DipElec205 code [S3]. In the calculation, we placed a hypothetical magnetic moment of 0.5μ_B at the center of the Mo_3O_{13} cluster.

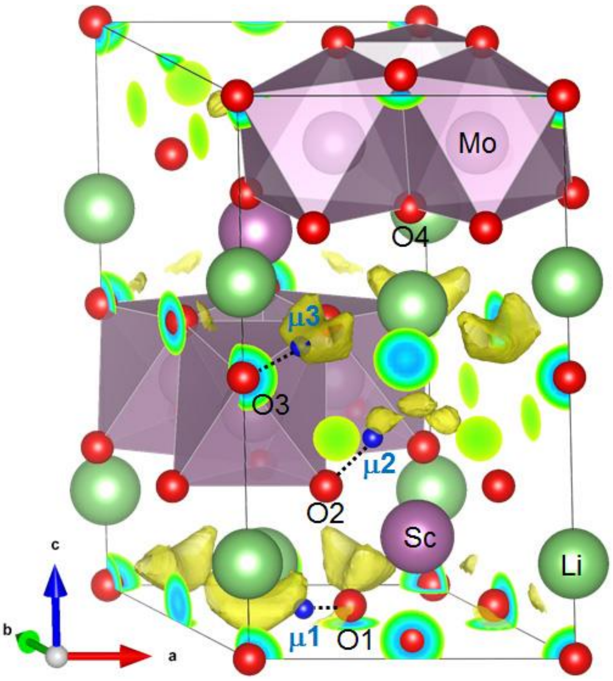

Figure S3. Schematic view of the distribution of local magnetic fields and muon stopping sites (μ1, μ2, and μ3) for $Li_2ScMo_3O_8$ illustrated by the VESTA software [S4]. Blue circles represent the muon sites whereas the local minima of electrostatic potential are expressed by yellow portions.

Table S3. Calculated local magnetic field δ_μ and nuclear dipolar field width Δ_μ at each muon site.

site	*x*	*Y*	*Z*	δ_μ (G)	Δ_μ (G)
μ1	0.297	0.297	0.05	235.9	3.198
μ2	0.5	0.25	0.36	209.5	2.864
μ3	0.226	0.113	0.54	169.1	3.702

Figure S4 shows the zero field- (ZF-)μSR spectrum of $Li_2ScMo_3O_8$ at 18.5 K. The spectrum was fitted by the following function: $A(t) = A_s G_z^{KT}(\Delta)e^{-\lambda t} + A_{BG}$ where A_s and A_{BG} are the positron decay asymmetries of the sample and background, $G_Z^{KT}(\Delta)$ the Kubo-Toyabe relaxation function [S5], and λ the muon relaxation rate in a motional narrowed line-shape region stems from the rapidly fluctuating electronic spins of the Mo_3O_{13} clusters. The spectrum was well fitted by the function as shown in Fig. S4 (the solid line). The fitted values are $\Delta = 0.155(2)$ μs^{-1} and $\lambda = 0.049(2)$ μs^{-1}. Therefore, the nuclear dipolar field at a muon site is found to be $\Delta_\mu = \sqrt{2}\Delta/\gamma_\mu = 2.57(3)$ G (γ_μ: muon gyromagnetic ratio). This experimental value coincides approximately with that of the calculated Δ_μ in μ2 site (see Table S3), which verifies our calculations on local magnetic fields.

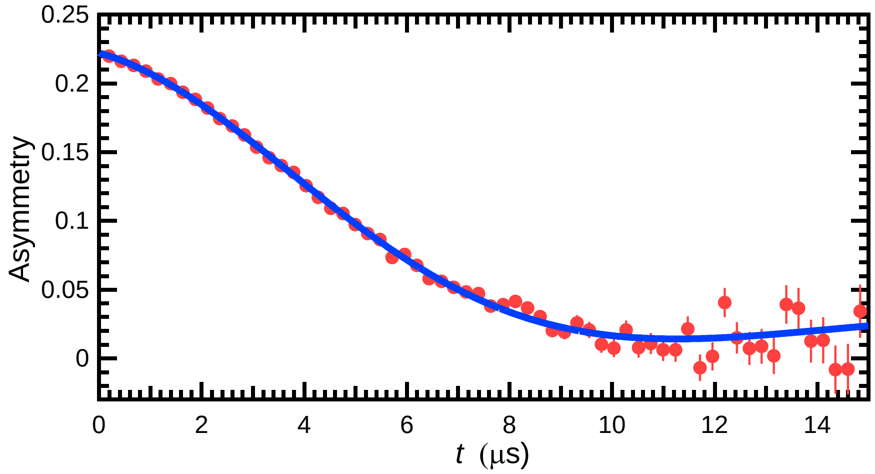

Figure S4. ZF-μSR time spectra of $Li_2ScMo_3O_8$ at 18.5 K. Solid line is fitting result.

References

[S1] Izumi, F. & Ikeda, T. A Rietveld-analysis program RIETAN-98 and its applications to zeolites. *Mater. Sci. Forum* **321-324**, 198-205 (2000).

[S2] Kresse, G. & Hafner, J. Ab initio molecular dynamics for liquid metals. *Phys. Rev. B* **47**, 558-561 (1993).

[S3] Kojima, K. M., Yamanobe, J., Eisaki, H., Uchida, S., Fudamoto, Y., Gat, I. M., Larkin, M. I., Savici, A., Uemura, Y. J., Kyriakou, P. P., Rovers, M. T. & Luke, G. M. Site-dilution in the quasi-one-dimensional antiferromagnet $Sr_2(Cu_{1-x}Pd_x)O_3$: reduction of Néel temperature and spatial distribution of ordered moment sizes. *Phys. Rev. B* **70**, 094402 (2004).

[S4] Momma, K. & Izumi, F. VESTA 3 for three-dimensional visualization of crystal, volumetric and morphology data. *J. Appl. Crystallogr.* **44**, 1272-1276 (2011).

[S5] Hayano, R. S., Uemura, Y. J., Imazato, J., Nishida, N., Yamazaki T. & Kubo, R. Zero- and low-field spin relaxation studied by positive muons. *Phys. Rev. B* **20**, 850-859 (1979).
